# Circulating microRNA-196a is an early gastric cancer biomarker

**DOI:** 10.18632/oncotarget.23126

**Published:** 2017-12-07

**Authors:** Tsung-Hsing Chen, Chieh Lee, Cheng-Tang Chiu, Yin-Yi Chu, Hao-Tsai Cheng, Jun-Te Hsu, Yung-Kuan Tsou, Ren-Chin Wu, Tse-Ching Chen, Nien-Chen Chang, Ta-Sen Yeh, Kwang-Huei Lin

**Affiliations:** ^1^ Department of Gastroenterology and Hepatology, Linkou Chang Gung Memorial Hospital, Chang Gung University College of Medicine, Taoyuan, Taiwan; ^2^ Graduate Institute of Clinical Medical Sciences, Chang Gung University, Taoyuan, Taiwan; ^3^ Department of Industrial Engineering and Management, Yuan Ze University College of Engineering, Chung-Li City, Taiwan; ^4^ Department of Surgery, Chang Gung Memorial Hospital, Linkou, Chang Gung University College of Medicine, Taoyuan, Taiwan; ^5^ Department of Pathology, Linkou Chang Gung Memorial Hospital, Chang Gung University College of Medicine, Taoyuan, Taiwan; ^6^ Graduate Institute of Biomedical Sciences, Chang Gung University, Taoyuan, Taiwan; ^7^ Liver Research Center, Chang Gung Memorial Hospital, Linkou, Taoyuan, Taiwan; ^8^ Research Center for Chinese Herbal Medicine, College of Human Ecology, Chang Gung University of Science and Technology Taoyuan, Taiwan

**Keywords:** microRNA-196a, precancerous gastric lesions, early gastric cancer, intestinal metaplasia, dysplasia

## Abstract

MicroRNA-196a (miRNA-196a) is associated with the development of gastric cancer and metastasis. Intestinal metaplasia and low- or high-grade dysplasia are considered to be precursors of intestinal type gastric cancer. Accordingly, we investigated the expression of plasma miRNA-196a as an early detection biomarker in precancerous gastric lesions and early cancer (pT1a/b), which is otherwise treated with endoscopic submucosal dissection. Our data showed that levels of circulating (plasma) miRNA-196a were higher in patients with precancerous lesions/early gastric adenocarcinoma than in healthy controls. The area under the receiver operating characteristic curve (AUC) for healthy controls vs. intestinal metaplasia was 0.9736; healthy controls vs. low-grade/high-grade dysplasia 0.9495; and healthy controls vs. early gastric cancer 0.9318. These results indicate that circulating miRNA-196a is a novel biomarker for detection of early gastric cancer and its precursor.

## INTRODUCTION

Gastric cancer (GC) remains a devastating disease, particularly in the advanced stage, due to the lack of biomarkers for early detection [1–3]. However, early intervention of precancerous gastric lesions and stage I GC leads to an excellent 5-year survival rate ≥ 95% [4]. Gastric cancer (GC) is histologically divided into two major types according to the Lauren classification: intestinal and diffuse. The former arises from atrophic gastritis (AG) and intestinal metaplasia (IM) [5–8]. IM is regarded as a “breaking point” [5–7] because *H. pylori* eradication results in histological improvement of gastric mucosa in AG (a precursor to IM) but not in IM. Currently, there is no clear consensus on gastric IM surveillance guidelines. However, early detection of GC may be achieved using an annual surveillance program if patients meet at least one of the following criteria: (1) IM extension > 20%; (2) presence of incomplete type IM; (3) first-degree relative with gastric cancer; or (4) smokers [9].

Gastroscopy is a standard method for GC screening, but patients are reluctant to undergo this invasive procedure. It often fails to detect precancerous gastric lesions and early GC because superficial mucosal lesions are easily missed even by optimum preparation and advanced technology such as image enhancement endoscopy (IEE) [10, 11], and conventional computed tomography has a disappointing sensitivity rate [12, 13]. Currently, carcinoembryonic antigen (CEA) and carbohydrate antigen 19-9 (CA19-9) were reported with positive detection at levels as low as 20.9% and 34.6%, respectively [14, 15]. There remains an urgent need for serological biomarkers for precancerous gastric lesions and early gastric cancer detection.

Sun et al. [16], Li et al. [17], and our previous study [18] have shown that expression of miRNA-196a is implicated in the carcinogenesis of gastric cancer. We further demonstrated that circulating miRNA-196a is strongly associated with metastatic gastric cancer progression [19], suggesting that miRNA-196a acts as a circulating biomarker for gastric cancer. In this study, we measured the expression of circulating miRNA-196a in precancerous gastric lesions and early gastric cancer to determine if circulating miRNA-196a can be employed as a useful biomarker in clinical practice.

## RESULTS

**Figure 1:**
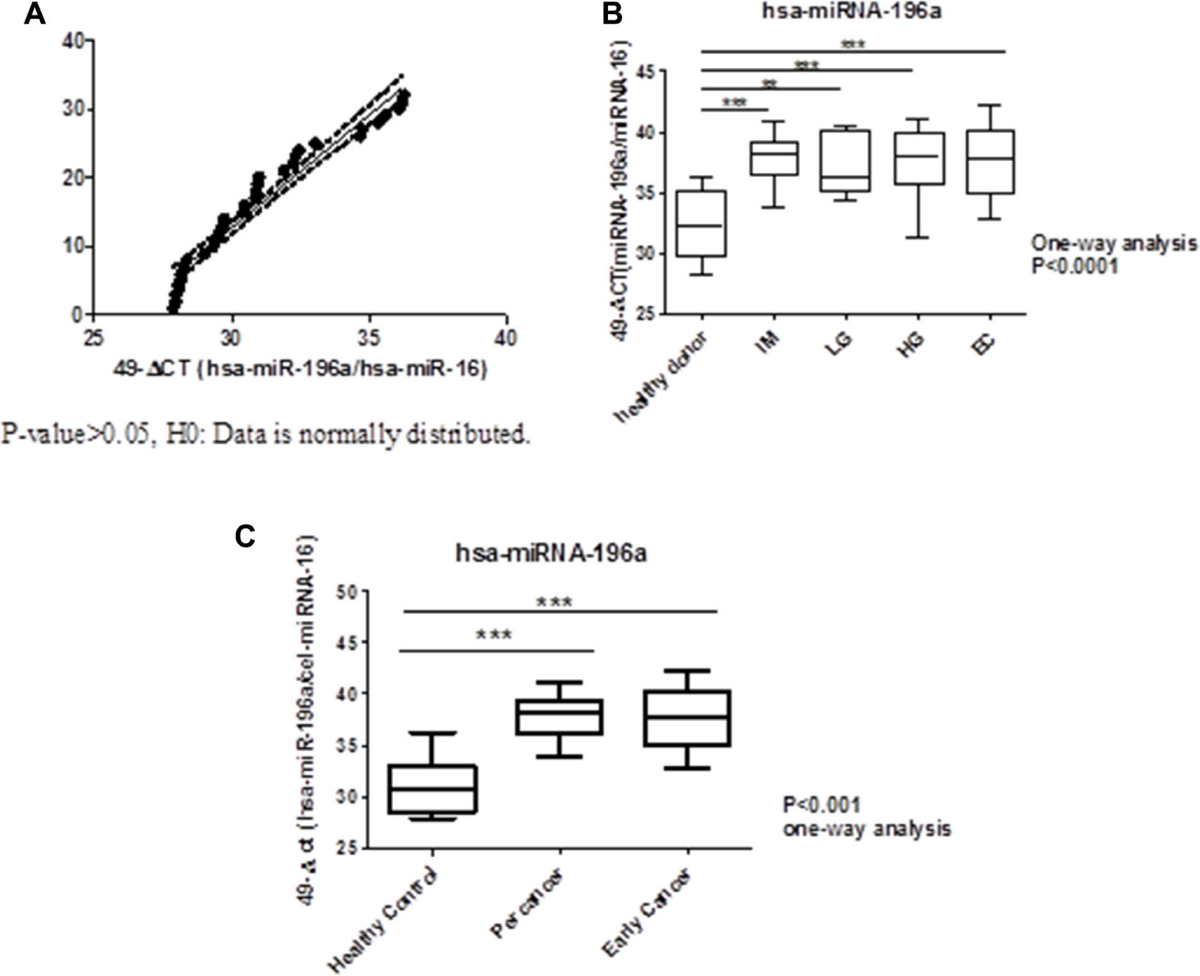
Normality test for healthy volunteers and boxplots of expression of miRNA196a in plasma. ^***^P-value < 0.001 ^**^P-value < 0.01 (**A**) Normality Test for Healthy Control. (**B**) Boxplots of Expression of miRNA-196a in Plasma for healthy donor (control), all stages of percancer and early cancer. (**C**) Boxplots of Expression of miRNA-196a in Plasma for healthy control, percancer, and early cancer. IM: intestinal metaplasia; LG: low-grade dysplasia; HG: high-grade dysplasia; EC: early cancer.

To verify circulating miRNA-196a expression as a biomarker of precancer and early gastric cancer, we recruited patients with intestinal metaplasia (*n* = 26), low/high-grade dysplasia (*n* = 13) and early gastric cancer (*n* = 11) who had undergone gastroscopic biopsies with pathological proof; while another 32 healthy volunteers served as controls. The mean ages of healthy controls, intestinal metaplasia, low/high- grade dysplasia (LGD/HGD), and early gastric cancer patients were 55 ± 12, 60 ± 9, 66 ± 14, and 64 ± 13, respectively. The expression of circulating miRNA-196a in health control, intestinal metaplasia, low/high – grade dysplasia and early gastric cancer detected by quantitative real-time polymerase chain reaction (qRT-PCR) were shown in Figure 1. It was found that expression of circulating miRNA-196a is lower in healthy control compared with all stages of pre-cancer (*p* < 0.001, *p* < 0.001, *p* < 0.001) and early cancer (*p* < 0.01); whereas there was no difference among precancers and early gastric cancer (*p* > 0.05).

**Figure 2:**
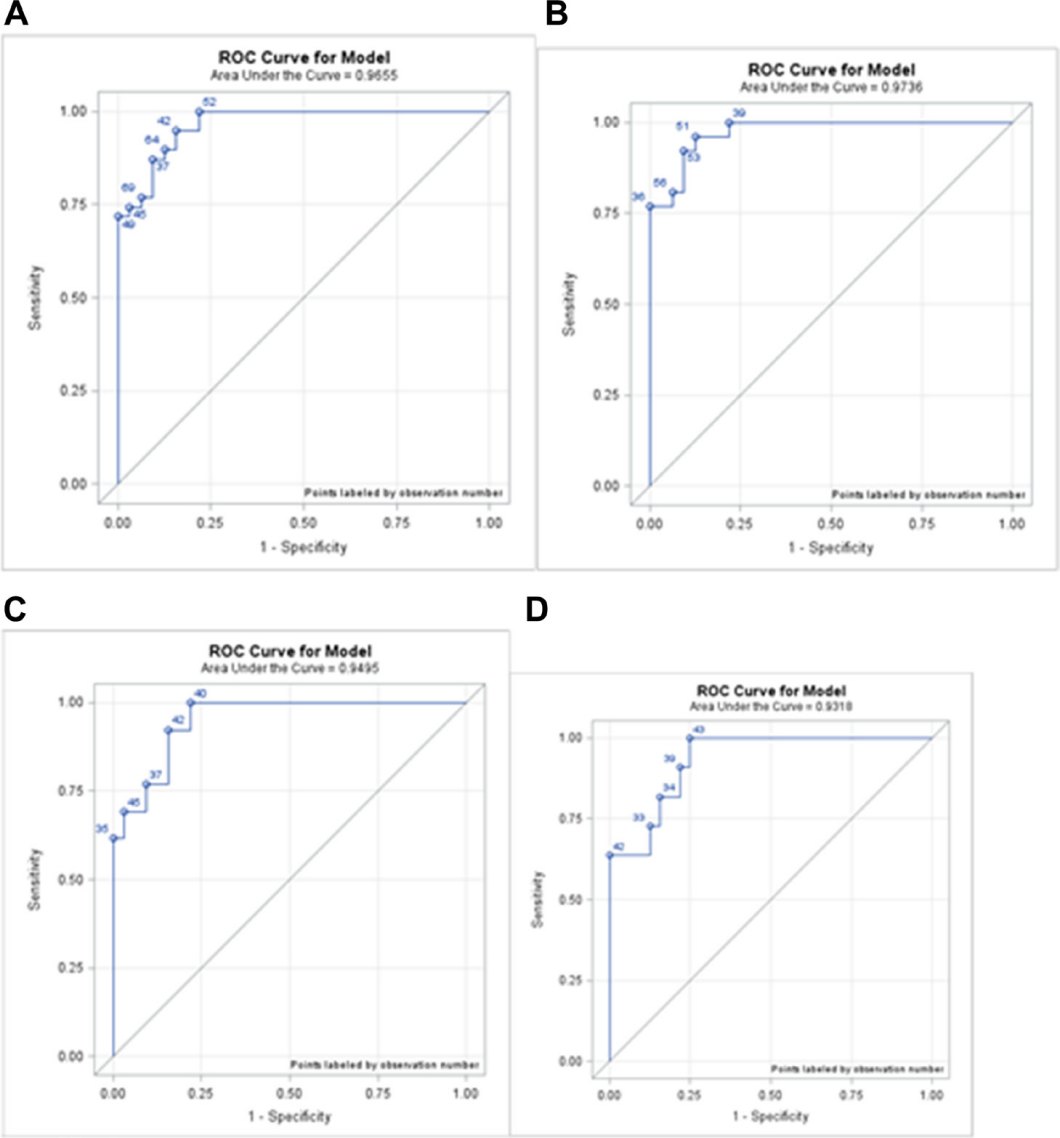
ROC curves for each phase (**A**) ROC curve of healthy subjects vs. IM, HGD, and LGD; AUC was 0.9655. (**B**) ROC curve of healthy subjects vs. IM; AUC was 0.9736. (**C**) ROC curve of healthy subjects vs. LGD and HGD; AUC was 0.9495. (**D**) ROC curve of healthy subjects vs. EC; AUC was 0.9318. IM: intestinal metaplasia; LGD: low-grade dysplasia; HGD: high-grade dysplasia; EC: early cancer.

The AUC for healthy controls vs. IM was 0.9736, the sensitivity and specificity were 96.15% and 87.50%, respectively; for healthy controls vs. LGD/HGD, the AUC was 0.9495, the sensitivity and specificity were 100.00% and 78.13%; for healthy controls vs. early GC the AUC was 0.9318, the sensitivity and specificity were 100.00% and 75.00% (Table 1 and Figure 2). The best cut-off points of miRNA-196a expression for each pre-cancer and early cancer stage are summarized as in Table 1. The logistic regression equations showed that the miRNA-196a is a significant predictor of whether a patient is healthy or at a specific pathological stage of the precancerous lesion (Table 2). To further demonstrate the effectiveness of miRNA-196a as a biomarker for GC, we implemented signal and detection theory to measure the distance between the signal and noise. The theory introduces a distance indicator discriminability index, d’, which is calculated by hit (correct prediction), miss, false alarm, and correct rejection rates. A larger d’ implies that the signal is further from the noise and suggests that miRNA-196a is a better pre-cancer or cancer indicator. The data for the hit, miss, false alarm and correct rejection rates of miRNA-196a expression in healthy volunteers vs. patients with pre-cancer and EC based on the cut-off points in Tables 1 and 2 are summarized in Table 3. The hit rate and the correct rejection rate are high. The miRNA-196a expression correctly identified all patients with LGD, HGD, and EC while keeping the false alarm rate relatively low. Similarly, miRNA-196a expression correctly identified over 96% of IM patients with a false alarm rate as low as 12.5%. These results indicate that miRNA-196a is a very promising indicator of pre-cancer as well as cancer occurrence.

**Table 1:** Summary of cutoff points for different phases of diagnostics

	AUC	Cutoff Points	95% CI (L, U)	Sensitivity	False Negative	Specificity	False Positive	*P*-Value
**H vs IM+LGD/HGD**	96.55%	36.2353	(35.282, 37.189)	74.36%	25.64%	96.88%	3.12%	<< 0.05
**H vs IM**	97.36%	35.3679	(34.306, 36.430)	96.15%	3.85%	87.50%	12.50%	<< 0.05
**H vs LGD/HGD**	94.95%	34.4784	(33.299, 35.658)	100.00%	0.00%	78.13%	21.87%	<< 0.05
**H vs EC**	93.18%	32.7643	(31.568, 33.961)	100.00%	0.00%	75.00%	25.00%	<< 0.05

Abbreviations: H: healthy control; IM: intestinal metaplasia; LGD: low-grade dysplasia; HGD: high-grade dysplasia; EC: early cancer; AUC: area under the curve.

**Table 2:** Summary of Logistics regression results for different phases of diagnostics

	Intercept	95% CI (L, U)	Estimated Coefficient of 49−eCT	95% CI (L, U)	*P*−Value
**H vs IM+LGD/HGD**	−40.2805	(−63.763, −16.798)	1.1519	(0.491, 1.813)	< 0.05
**H vs IM**	−46.1239	(−77.975, −14.273)	1.2992	(0.412, 2.186)	< 0.05
**H vs LGD/HGD**	−36.9854	(−64.962, −9.019)	1.0332	(0.247, 1.818)	< 0.05
**H vs EC**	−25.1915	(−41.229, −9.154)	0.7012	(0.247, 1.156)	< 0.05

Abbreviations: H: healthy control; IM: intestinal metaplasia; LGD: low-grade dysplasia; HGD: high-grade dysplasia.

**Table 3:** Summary of indicators from signal and detection theory for healthy control vs. patients in precancerous and early cancer stages

Stages	Cutoff point	95% CI (L, U)	Hit	False Alarm	Miss	Correct Rejection	d′
H vs. IM+LGD/HGD	36.2353	(35.282, 37.189)	74.36%	3.13%	25.64%	96.88%	2.493
H vs IM	35.3679	(34.306, 36.430)	96.15%	12.50%	3.85%	87.50%	2.919
H vs LGD/HGD	34.4784	(33.299, 35.658)	100.00%	21.88%	0.00%	78.13%	5.076
H vs EC	32.7643	(31.568, 33.961)	100.00%	25.00%	0.00%	75.00%	4.974

Abbreviations: H: healthy control; IM: intestinal metaplasia; LGD: low-grade dysplasia; HGD: high-grade dysplasia; EC: early cancer.

**Figure 3:**
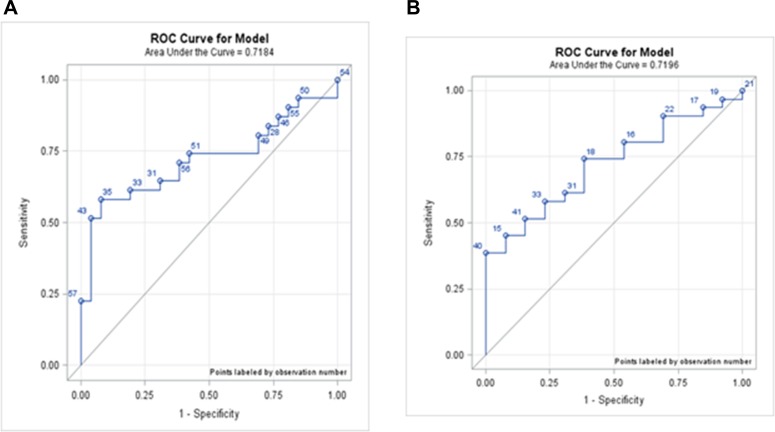
ROC curves for each phase (**A**) ROC curve of IM vs. EC; AUC was 0.7184. (**B**) ROC curve of LGD/HGD, vs. EC; AUC was 0.7196 IM: intestinal metaplasia; LGD: low-grade dysplasia; HGD: high-grade dysplasia; EC: early cancer.

However, miRNA-196a expression did not significantly differ between pre-cancer patients and cancer patients. The AUC of IM vs. early GC was 0.7184, and LGD/HGD vs. early GC was 0.7196, both greater than 0.5 (Figure 3). The d’ of IM vs. early GC was 1.222, and LGD/HGD vs. early GC was 4.0120, both greater than 0. We concluded that the miRNA-196a could weakly differentiate precancerous lesions from early cancer. Even though both the AUC and d’ indicate that miRNA-196a could potentially serve as an indicator for pre-cancer or cancer, in practice, the hit rates were relatively low (Table 4).

**Table 4:** Summary of indicators from signal and detection theory for precancerous and early cancer stages

	Cutoff point	95% CI (L, U)	Hit	False Alarm	Miss	Correct Rejection	d′
IM vs LGD/HGD	35.8114	(35.208, 36.415)	30.77%	7.69%	69.23%	92.31%	0.9240
IM vs EC	35.7565	(34.993, 36.520)	58.06%	7.69%	41.94%	92.31%	1.2220
LGD/HGD vs EC	34.2892	(33.344, 35.234)	38.71%	0.00%	61.29%	100.00%	4.0130

Abbreviations: H: healthy control; IM: intestinal metaplasia; LGD: low-grade dysplasia; HGD: high-grade dysplasia; EC: early cancer.

## DISCUSSION

Few studies have reported the detection of precancerous gastric lesions, particularly in the plasma. GC usually has a poor outcome as a result of the delay in diagnosis. Nevertheless, progression to cancer can be prevented if mucosal changes (e.g. AG or IM) are detected as early as possible. Endoscopic submucosal dissection can be done to excise the lesion once early gastric cancer or high-grade dysplasia is found while preserving the organ and promoting a good quality of life [20, 21]. At early stages most patients are asymptomatic, and the cancer is hard to detect, so it is challenging to prevent its progression. Although gastroscopy is a standard screening tool for gastric lesions, many patients consider it too uncomfortable. Thus, a liquid biopsy is more suitable for these asymptomatic patients, followed by gastroscopy to find those at risk.

The most pressing challenge is the identification of patients at high risk of diffuse or intestinal type GC. The latter is preceded by a sequence of precursor steps, which (colonic-type intestinal metaplasia) or small intestinal mucosa [22]. In our study, miRNA-196a expression is lower in healthy control compared with all stages of precancer, which might partially explain why IM is considered a “breaking point.”

In our previous study, we found that circulating miRNA-196a/b could be used as a novel biomarker for metastatic gastric cancer prediction [18]. Here, we verified that the expression level of circulating miRNA-196a is a predictive biomarker for precancerous gastric lesions and GC. We found that miRNA-196a expression levels can distinguish healthy controls from those with precancerous gastric lesions (Table 1&2 and Figure 1). The sensitivity and specificity of healthy controls vs. the IM+LGD/HGD group were 74.36% and 96.88%; healthy controls vs. IM 96.15% and 87.50%; healthy controls vs. LGD/HGD 100.00% and 78.13%; healthy controls vs. EC 100.00% and 75.00%. Our results show that circulating miRNA-196a can distinguish patients with IM, LGD, HGD and early GC from healthy controls, and especially healthy participants from those subjects with gastric dysplasia and early cancer.

## MATERIALS AND METHODS

### Subjects

Plasma samples were collected from 50 patients (14 women and 36 men) from 2014 to 2016 at Chang Gung Memorial Hospital. The participants were divided into four subgroups, IM, low-grade dysplasia (LGD), high-grade dysplasia (HGD), and early gastric cancer (EC), and independently interpreted by two senior pathologists. A total of 32 healthy volunteers (18 women and 14 men) were enrolled from Chang Gung Memorial Hospital healthcare center and served as controls. These healthy volunteers were verified by negative results of routine blood test, chest radiographs, endoscopy examination (white light endoscopy), ultrasonography, cancer screening tests, and computed tomography /positron emission tomography scan. IEE was performed to validate patients with or without gastric mucosal change. The study was approved by the Institutional Review Board (IRB No. 103-2095C) of Chang Gung Memorial Hospital, and all participants gave informed consent.

### Quantification of plasma miRNA-196a

The concentrations of miRNA from plasma samples were quantified using a NanoDrop 1000 spectrophotometer (Nanodrop Technologies Inc., Wilmington, DE, USA.). Circulating miRNA-196a expression was determined by quantitative real-time polymerase chain reaction (qRT-PCR), as described previously, while miR-16 served as an internal control [18, 23–25]. Fluorescence emitted by SYBR green was assayed using the ABI Prism 7500 Fast Real-Time PCR system (Life Technologies). For plasma analysis, after normalization to the spike-in control (miR-16), we calculated 49-ΔCt to compare the values between patients and healthy control.

### Statistical analysis

The *t*-test and a one-way analysis of variance (ANOVA) were used for pair-wise comparison of the plasma miRNA-196a expression in different stages of pre-cancer and early cancer. The Kolmogorov-Smirnov test with α = 0.05 was used to verify the normality of healthy control samples. The receiver operating characteristic (ROC) curve and area under the curve (AUC) were used to assess the potential of using the plasma expression of miRNA-196a as a diagnostic tool for EC and precancerous lesions, such as incomplete and complete IM, LGD, and HGD. Youden's Index was employed to determine the theoretical best cut-off points for miRNA-196a expression between healthy volunteers, incomplete and complete IM (hence after IM), LGD, HGD, and EC. Signal detection theory was used to find the best practical cut-off points for miRNA-196a expression from IM to early cancer and LGD/HGD to early cancer. The AUC and d’ were reported as indicators of the strength of the model. Signal and detection theory was implemented to determine the best practical cut-off points along with the probability of hit, miss, false alarm, and correct rejection. Descriptive data analyses were done using Prism 5 (GraphPad Software Inc., San Diego, CA, USA). Statistical analyses including ROC and signal detection theory were conducted using SAS 9.4 (SAS Institute Inc., Cary, NC, USA).

## CONCLUSIONS

Circulating miRNA-196a is an effective biomarker that could afford patients with gastric dysplasia or early GC the chance to receive minimally invasive procedures to mitigate the disease progression. For example, we suggest that asymptomatic subjects with a high risk of developing gastric cancer could be screened by blood sampling for miRNA-196a. A follow-up with intensive gastroscopy (best combined with IEE) would be strongly advised for patients with significant changes in the circulating miRNA-196a level. Given that the number of patients with dysplasia and the total number of our study group was small, a large-scale study of circulating miRNA-196a as a biomarker for gastric pre-cancer detection is necessary.
